# Mantle flow underneath the South China Sea revealed by seismic anisotropy

**DOI:** 10.1093/nsr/nwad176

**Published:** 2023-06-16

**Authors:** Fansheng Kong, Rui Gao, Stephen S Gao, Kelly H Liu, Weiwei Ding, Xiongwei Niu, Aiguo Ruan, Pingchuan Tan, Jianke Fan, Shaoping Lu, Zhengyi Tong, Liqun Cheng, Wenfei Gong, Yanghui Zhao, Jiabiao Li

**Affiliations:** Key Laboratory of Submarine Geosciences, Second Institute of Oceanography, Ministry of Natural Resources, Hangzhou310012, China; Southern Marine Science and Engineering Guangdong Laboratory, Zhuhai519082, China; Geology and Geophysics Program, Department of Geosciences and Geological and Petroleum Engineering, Missouri University of Science and Technology, Rolla, MO 65409, USA; Southern Marine Science and Engineering Guangdong Laboratory, Zhuhai519082, China; School of Earth Sciences and Engineering, Sun Yat-sen University, Zhuhai519082, China; Geology and Geophysics Program, Department of Geosciences and Geological and Petroleum Engineering, Missouri University of Science and Technology, Rolla, MO 65409, USA; Geology and Geophysics Program, Department of Geosciences and Geological and Petroleum Engineering, Missouri University of Science and Technology, Rolla, MO 65409, USA; Key Laboratory of Submarine Geosciences, Second Institute of Oceanography, Ministry of Natural Resources, Hangzhou310012, China; Southern Marine Science and Engineering Guangdong Laboratory, Zhuhai519082, China; Key Laboratory of Submarine Geosciences, Second Institute of Oceanography, Ministry of Natural Resources, Hangzhou310012, China; Southern Marine Science and Engineering Guangdong Laboratory, Zhuhai519082, China; Key Laboratory of Submarine Geosciences, Second Institute of Oceanography, Ministry of Natural Resources, Hangzhou310012, China; Southern Marine Science and Engineering Guangdong Laboratory, Zhuhai519082, China; Key Laboratory of Submarine Geosciences, Second Institute of Oceanography, Ministry of Natural Resources, Hangzhou310012, China; Southern Marine Science and Engineering Guangdong Laboratory, Zhuhai519082, China; Key Laboratory of Marine Geology and Environment, Institute of Oceanology, Chinese Academy of Sciences, Qingdao266071, China; Southern Marine Science and Engineering Guangdong Laboratory, Zhuhai519082, China; School of Earth Sciences and Engineering, Sun Yat-sen University, Zhuhai519082, China; Key Laboratory of Submarine Geosciences, Second Institute of Oceanography, Ministry of Natural Resources, Hangzhou310012, China; Key Laboratory of Submarine Geosciences, Second Institute of Oceanography, Ministry of Natural Resources, Hangzhou310012, China; Key Laboratory of Submarine Geosciences, Second Institute of Oceanography, Ministry of Natural Resources, Hangzhou310012, China; Key Laboratory of Submarine Geosciences, Second Institute of Oceanography, Ministry of Natural Resources, Hangzhou310012, China; Southern Marine Science and Engineering Guangdong Laboratory, Zhuhai519082, China; Key Laboratory of Submarine Geosciences, Second Institute of Oceanography, Ministry of Natural Resources, Hangzhou310012, China; Southern Marine Science and Engineering Guangdong Laboratory, Zhuhai519082, China

**Keywords:** mantle flow, South China Sea, Tibetan Plateau, seismic anisotropy, shear wave splitting

## Abstract

It has long been established that plastic flow in the asthenosphere interacts constantly with the overlying lithosphere and plays a pivotal role in controlling the occurrence of geohazards such as earthquakes and volcanic eruptions. Unfortunately, accurately characterizing the direction and lateral extents of the mantle flow field is notoriously difficult, especially in oceanic areas where deployment of ocean bottom seismometers (OBSs) is expensive and thus rare. In this study, by applying shear wave splitting analyses to a dataset recorded by an OBS array that we deployed between mid-2019 and mid-2020 in the South China Sea (SCS), we show that the dominant mantle flow field has a NNW–SSE orientation, which can be attributed to mantle flow extruded from the Tibetan Plateau by the ongoing Indian–Eurasian collision. In addition, the results suggest that E–W oriented flow fields observed in South China and the Indochina Peninsula do not extend to the central SCS.

## Teaser

New geophysical measurements, obtained from ocean-bottom seismometers, suggest that the continental collision occurring along the Himalayas exerts a significant influence on the mantle flow field beneath the South China Sea.

## INTRODUCTION

One of the cornerstones of the theory of plate tectonics is the realization and characterization of the asthenosphere and the roles that it plays in facilitating and modulating plate motion [1]. Specifically, this mechanically weak layer allows relative movements between rigid plates along plate boundaries and provides driving or resistant forces for plate motions, during which processes the Earth's surface is continuously shaped through orogeny, volcanism, earthquakes, and other geological processes [2–4]. Delineating the direction, strength, and spatial distributions of plastic flow within the asthenosphere may, therefore, offer essential constraints on plate dynamics [2–5]. In areas adjacent to divergent plate margins (ocean ridges), the horizontal component of the asthenospheric flow is mainly influenced by plate motions [2] and/or thermal convection beneath mid-ocean ridges [5]. In convergent margins where an oceanic plate underthrusts another oceanic plate or continental mass, flow in the asthenosphere largely takes the form of corner flow induced by the shear drag associated with the subducting slab [6–8]. Ambiguities exist in terms of how far such a flow system extends laterally. In another type of convergent boundary associated with continental–continental collision, such as the ongoing collision between the Indian continent and the Tibetan Plateau, it is suggested that the asthenosphere may accommodate the collision by being extruded into adjacent regions [9–12]. However, how far the extruded asthenospheric flow can reach remains unclear.

**Figure 1.**
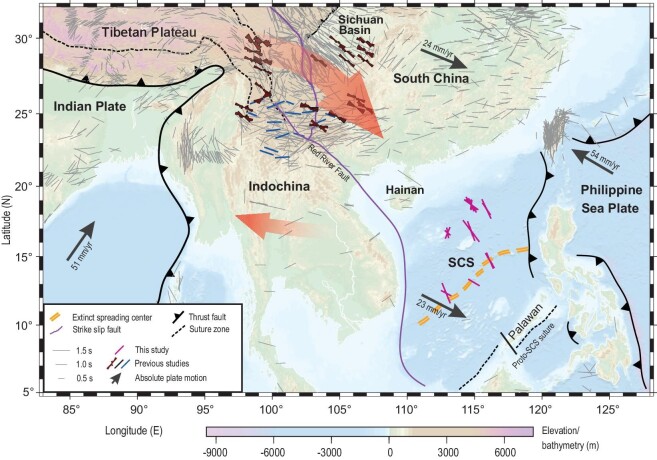
Topographic map of the study area and adjacent subduction zones. The black arrows represent the absolute plate motion directions obtained using the NNR-MORVEL56 model with numbers denoting the velocity [29]. The black bars show the station-averaged apparent shear wave splitting measurements from previous studies obtained at http://splitting.gm.univ-montp2.fr/DB/public/searchdatabase.html, and the red ones denote the individual measurements obtained in this study. The orientation of the bars characterizes the fast orientation of seismic anisotropy, and the length is proportional to the size of the splitting time. The blue bars [13] and rose diagrams [9] represent the fast orientations of anisotropy related to asthenospheric flow from previous investigations in regions with complex anisotropy where station-averaged apparent splitting measurements cannot effectively characterize the complex anisotropic signature. The red arrow on the North side exhibits the extruded mantle flow from the Indian-Eurasian collision zone and the southern red arrow stands for the E–W oriented flow field associated with the subducting Indian slab beneath the Indochina Peninsula. SCS: South China Sea.

The South China Sea (SCS) is an ideal locale to probe the lateral extents of the asthenospheric flow systems driven by plate subduction and continental collision. To the West, the Indian plate is subducting beneath the Indochina Peninsula and adjacent regions. Subduction of the Indian plate is considered to drive the E–W oriented corner flow system underneath the Indochina Peninsula, although the eastern boundary of the corner flow field is not clear due to a paucity of relevant observations in the SCS [8,13,14]. To the northwest of the SCS, the collision between the Indian and Eurasian plates resulted in lithospheric extrusions starting at the Eocene, causing the Indochina and South China blocks to move hundreds of kilometers to the East or SE relative to the Tibetan Plateau [15]. Growing lines of evidence show that the extrusion also transports asthenospheric materials from the central Tibetan Plateau mostly eastward, which are blocked by the cone-shaped lithospheric root of the Sichuan Basin and flow divergently along the craton margins [9–12,16]. Recent seismological studies indicate that a branch flows southeastward along the boundary between the southeastern Tibetan Plateau and the South China block [9,10] (Fig. 1). However, whether the extruded asthenospheric materials continue flowing southeastward and reach the SCS remains vague.

### Multi-episode spreading in the South China Sea

The SCS, one of the largest marginal seas in the West Pacific [17], is considered to have started spreading in the early Oligocene and ended in the mid-Miocene [18–20], during which period the rise of the Himalayas significantly accelerated [21]. Previous studies suggest that the SCS experienced multiple spreading episodes characterized by different spreading directions [18–20,22] and accompanied by more than one southward ridge jump [18,23]. The first episode of spreading opened the NW and East sub-basins in a nearly N–S direction [19,22], and the following stages with a spreading direction of NW–SE involved the East and SW sub-basins [22]. Although a large number of studies on the formation and evolution of the SCS have been conducted over the past several decades [22–24], the vast majority of the geophysical explorations have been aimed at delineating the structures and deformations in the crust and shallow lithospheric mantle. By contrast, conclusive investigations regarding the existence, direction, and driving mechanisms of the flow field in the asthenosphere beneath the SCS remain lacking [25].

### Previous investigations of asthenospheric flow underneath the South China Sea

The mantle flow fields in the SCS are highly debated in terms of the dominant flow direction and the primary driving forces. Previously proposed models include 1) a southeastward mantle flow system originated from the Tibetan Plateau which enters the SCS from the NW margin and then turns to the West [26]; 2) a northeastward flow system beneath the SCS driven by a broad mantle upwelling zone that is associated with the slabs subducted from the East and South [27]; and 3) a flow system induced by plate motion [28], which is moving at an azimuth of about 117° clockwise from the North at a rate of 24 mm/year in central SCS based on the NNR-MORVEL56 model [29]. One of the primary reasons for the ambiguity and controversy in the mantle flow field of the SCS is that almost all of the passive source seismic data utilized in the previous investigations are obtained from the nearby onshore seismic stations [28,30,31], which cannot sufficiently sample the upper mantle of the SCS via body waves [26,28].

### Seismic anisotropy and shear wave splitting

The plastic mantle flow can be readily constrained and characterized based on the strength and orientation of seismic azimuthal anisotropy [32,33]. In the upper mantle, simple shear induced by differential motions between the asthenosphere and the overlying lithosphere can produce lattice preferred orientation of anisotropic minerals, primarily olivine [34], which is widely regarded as the cause of upper mantle seismic anisotropy with the fast axis parallel to the shear direction under typical upper mantle conditions [33]. The anisotropic signature can be fossilized in the lithosphere near a mid-ocean ridge during seafloor spreading with fast axes in accordance with the spreading direction [35].

As demonstrated by numerous studies [36–39], splitting analysis of core-mantle-boundary refracted shear waves, i.e. PKS, SKKS, and SKS phases (collectively referred to as XKS hereafter), is arguably the most widely used approach to constrain seismic azimuthal anisotropy and characterize plastic flow fields in the upper mantle. When a shear wave travels sub-vertically through an anisotropic layer with a horizontal axis of symmetry, it splits into two shear waves characterized by orthogonal polarization directions and different propagating seismic velocities [36]. The time delay (the splitting time or δt) that is accumulated owing to the different velocities when passing through the anisotropic layer, positively correlates with the thickness and/or the strength of seismic anisotropy, while the polarization direction of the fast shear wave (the fast orientation or ϕ) is constantly used to infer the orientation of seismic anisotropy [32]. If simple anisotropy, i.e. a single layer of transverse isotropy with a horizontal axis of symmetry, is present, the fast orientations of the individual XKS splitting measurements would be invariant with respect to the arriving azimuth of the seismic ray path (the back azimuth), and therefore the station-averaged shear wave splitting parameters can be used to effectively constrain anisotropic signatures [32]. Any significant departure from this simple model is referred to as complex anisotropy, with the most common form comprising two layers with non-parallel-and-non-perpendicular fast orientations. The resulting splitting parameters under a two-layered anisotropy model are expected to exhibit a 90° periodic variation when plotted against the back azimuth [39].

To enhance the spatial coverage of passive source seismic data and provide additional constraints on seismic anisotropy and mantle flow geometry beneath the SCS, 46 broadband ocean-bottom seismometers (OBSs) were deployed in the SCS basin in mid-2019, among which 17 were recovered in mid-2020 with a recording period of approximately nine months (Fig. S1). Possible factors accounting for the low data recovery rate can be found in the Supplementary data. Among the 17 recovered OBSs, eight experienced failures of at least one of the three channels and were removed from the shear wave splitting analysis. Seismic data from a previous array comprising seven OBSs with a recording period of approximately three months [40] (Fig. S1) are also used in this study. The obtained broadband seismic data from the 16 OBSs provided a unique opportunity to systematically investigate the anisotropic structure and mantle flow field beneath the SCS.

## RESULTS

**Figure 2.**
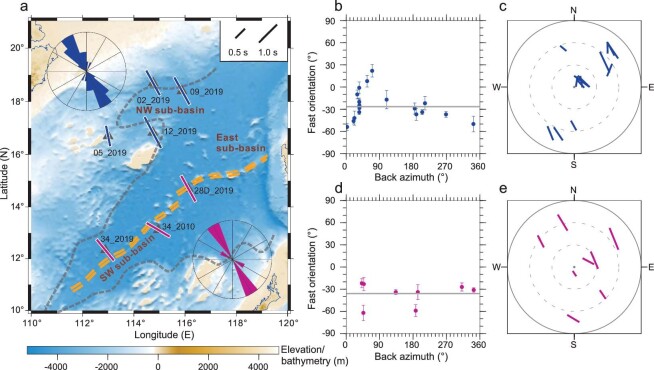
(a) Station-averaged shear wave splitting parameters plotted at the OBS deployment locations. The measurements from the OBSs located at the NW sub-basin are shown in blue, while those adjacent to the fossil spreading ridge of the SW sub-basin are in red. The rose diagrams exhibit the individual fast orientations in the two sub-basins. The double yellow dashed lines indicate the extinct spreading ridge axis. OBS names are marked near deployment locations with the latter part denoting the year of deployment. (b, d) Individual fast orientations from the NW and SW sub-basins, respectively, plotted against the back azimuth with the grey lines representing the circular means. (c, e) Same as (b, d) but in a stereographic plot. The length of the bars is proportional to the size of the splitting time, while its location is associated with the back azimuth and epicentral distance.

After data preprocessing and horizontal component corrections (see Supplementary data for details; Fig. S2), a total of 26 pairs of well-defined XKS shear wave splitting measurements (the fast orientation and splitting time) from 25 teleseismic events (Fig. S3 and Supplementary Table S1) were obtained at seven of the 16 OBSs (Figs 1 and 2) using the transverse energy minimization method [32]. To ensure reliability, all the resulting splitting measurements were manually checked. Errors in the individual splitting measurements were estimated using the inverse *F* test and represent the 95% confidence level [32], which range from 3° to 12.5° for the fast orientation and 0.07 to 0.45 s for the splitting time. Both are within the ranges for typical well-defined measurements [2,41]. Examples of individual splitting measurements are shown in Fig. S4.

To obtain a first-order overview of the spatial patterns of the resulting splitting parameters and to identify the existence or absence of complex anisotropy, we combined the measurements from nearby OBSs located in or near the same sub-basin (Fig. 2). The splitting measurements from the OBSs in the vicinity of the axial fossil ridge of the SW sub-basin are azimuthally invariant (Figs 2 and S5) with a circular mean of 144.1° ± 14.6° (clockwise from the North) for the fast orientation and an arithmetic average of 1.11 ± 0.16 s for the splitting time. The circular mean of the resulting fast orientations differs from the absolute plate motion (APM) direction by ∼27° and is consistent with the fossil seafloor spreading direction in the SW sub-basin [18,22].

The measurements from the OBSs deployed on the NW sub-basin seafloor and adjacent areas, which have a circular mean of 153.4° ± 19.5° for the fast orientations and an arithmetic mean of 1.16 ± 0.11 s for the splitting times, are characterized by an azimuthally variant pattern (Figs 2 and S6). The azimuthal variation is generally consistent with the presence of a two-layered anisotropy model with non-parallel-and-non-perpendicular fast orientations. Since numerical experiments [13] suggest that when the splitting times in a two-layered anisotropy model are significantly different from each other, azimuthal variations of the splitting parameters are expected to occur in a narrow back azimuth range, a pattern that generally fits the observations in the NW sub-basin (Fig. 2). In such cases, station-averaged splitting parameters are similar to those of the anisotropic layer with the larger splitting time. Therefore, in the following, we used station-averaged splitting parameters as a first-order representation of the anisotropic structure for both the SW and NW sub-basins. Given the apparent azimuthal variation observed in the NW sub-basin, anisotropy models composed of two layers with non-parallel-and-non-perpendicular fast orientations (Figs S7 and S8) are also discussed.

## DISCUSSION

### Anisotropy fossilized in the lithosphere during seafloor spreading

**Figure 3.**
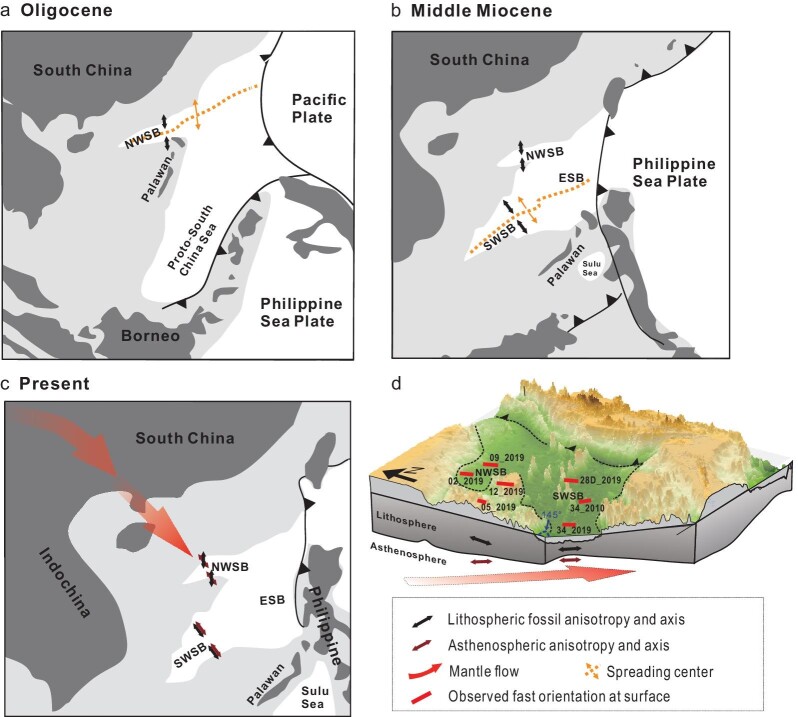
Schematic diagram of the preferred model for the origins of anisotropy accounting for the observed XKS splitting measurements. Anisotropy in the lithosphere (a, b) is related to the seafloor spreading in the Oligocene for the NW sub-basin and the spreading in the mid-Miocene for the SW sub-basin. Anisotropy originated in the asthenosphere (c) plays a major role in accounting for the observed splitting measurements and is induced by the present-day mantle flow field associated with the Indian-Eurasian collision (red arrows). (d) Schematic illustration of the model in depth domain. NWSB: NW sub-basin. SWSB: SW sub-basin. ESB: East sub-basin.

It has been well documented that in the vicinity of a mid-ocean ridge, corner flow beneath spreading centers would align the fast axis of olivine in accordance with the seafloor spreading direction, resulting in seismic azimuthal anisotropy that is ‘fossilized’ in the lithospheric mantle during the formation of a new oceanic plate [35]. Previous magnetic studies [18,19,22] suggest that the spreading direction of the NW sub-basin in the Oligocene is ∼175° clockwise from the North and that of the SW sub-basin that occurred in the mid-Miocene is ∼145°. Under this model, fossilized anisotropy in the lithosphere, if present, would have a fast orientation of ∼175° in the NW sub-basin and ∼145° in the SW sub-basin (Fig. 3). Assuming a typical anisotropy strength of 4% in the upper mantle [42], a lithospheric mantle of ∼40-km thickness in the NW sub-basin [43] may result in a splitting time of ∼0.35 s [32]. For the SW sub-basin, which has a lithosphere as thin as ∼20 km [44], the contribution to the splitting time is ∼0.15 s. Therefore, the expected splitting times for both sub-basins are significantly smaller than the observed values, suggesting that the observed NNW–SSE oriented anisotropy is mostly from the sub-lithospheric mantle. Combining this observation and the arguments against a mantle transition zone and lower mantle origin of the observed anisotropy (see Supplementary data for details) indicates that the observed anisotropy is mostly from the asthenospheric mantle.

### Extent of subduction-induced mantle flow associated with the Indian slab

The aforementioned realization that the observed anisotropy is largely from NNW–SSE oriented mantle flow system beneath the lithosphere provides an important constraint on the lateral extent of the E–W oriented corner flow system associated with the oblique subduction of the Indian slab beneath the Indochina Peninsula. Numerous shear wave splitting studies [8,13,14] sufficiently demonstrated the existence of an E–W oriented flow system from near the Burma subduction zone to the eastern coast of the Indochina Peninsula (Fig. 1) and attributed this flow system to the subduction of the Indian slab beneath the Peninsula. Our splitting measurements have a NNW–SSE orientation and imply that the E–W flow system does not extend to the central SCS. A receiver function stacking study [45] suggests that the slab reaches the 660-km discontinuity near the eastern edge of the Peninsula and induces upwelling of high temperature lower mantle materials in the western SCS. Combining this observation and our shear wave splitting measurements, we propose that subduction induced corner flow does not extend beyond the location where the slab penetrates to the lower mantle.

### Present-day mantle flow field associated with the Indian–Eurasian collision

Assuming a stationary asthenosphere, the motion of the overlying lithosphere can induce simple shear which would generate azimuthal anisotropy with a resulting fast orientation parallel to the plate motion direction. However, the APM-driven simple shear may not be the dominating factor in producing the anisotropy in the two sub-basins for the following reasons. First, the plate motion rate in the study area is about 22 to 24 mm/year, which is lower than the threshold value (30 mm/year) required for the APM to possess a dominating process [46]. Second, the observed fast orientations differ from the APM direction based on the NNR-MORVEL56 model [29] by a non-negligible angular difference of 36° for the NW sub-basin and 27° for the SW sub-basin (Fig. 2). Considering the azimuthal variations of the individual splitting parameters (Figs 2 and S6) in the NW sub-basin, a two-layered anisotropy model was constructed with the upper layer related to the aforementioned fossilized anisotropy in the lithosphere and the lower layer associated with the APM. For the fast orientation, some of the observed values (e.g. the two in the azimuthal range of 50°–70°) differ significantly from the predicted values (Fig. S7). By contrast, the fitting improves substantially for a two-layered anisotropy model with a lower layer fast orientation that is parallel to the NNW–SSE extruded flow (Fig. S8) driven by the Indian–Eurasian collision as discussed below. Third, if APM-induced anisotropy is the main source, the ocean–continental transitional zone where an abrupt change of lithosphere thickness is present [43] may modulate mantle flow and result in a fast orientation that is parallel to the edge of the NW sub-basin [16,47]. Such variation in the individual fast orientations was not observed in this study (Fig. 2).

Our preferred model (Figs 3 and S8) for the anisotropy originating in the sub-lithospheric mantle of the two sub-basins invokes a mantle flow field that is southeastward or in the opposite direction (as shear wave splitting determines the orientation and not the direction of a flow system). Previous studies in the southeastern Tibetan Plateau and adjacent regions suggest the presence of southeastward mantle flow in the asthenosphere [9–12], which is believed to be from the Tibetan Plateau in response to the Indian–Eurasian plate collision and deflected by the cone-shaped lithospheric root of the Sichuan Basin. Our measurements suggest that the extruded mantle flow probably continues southeastward and reaches the central SCS (Fig. 3). The absence of clear azimuthal dependence of the individual splitting parameters in the SW sub-basin suggests that anisotropy in the lithosphere, which reflects frozen-in fabric associated with ancient seafloor spreading, aligns with the collision-extruded flow system at the present time [18] and can be approximated by a model of single-layered anisotropy. By contrast, the splitting parameters obtained in the NW sub-basin can be explained by a two-layered anisotropy model (Fig. S8), in which the top layer has a fast orientation that is parallel to the ancient seafloor spreading direction of ∼175° [18,19,22], and the lower layer has a fast orientation that is associated with the NNW–SSE extruded flow, resulting in the azimuthal dependence of the individual splitting parameters.

An interesting observation is that in southern China, the fast orientations are mostly E–W which is generally regarded to be induced by either subduction or APM. The apparent discontinuity of the NNW–SSE extruded flow system in the region between the area with the rose diagrams in Fig. 1 and the SCS can be explained by the co-existence of two flow systems with E–W and NNW–SSE fast orientations. In the area where E–W fast orientations are observed, the E–W anisotropy-forming processes (e.g. slab subduction and APM) have a much greater strength than the NNW–SSE flow system. The latter system becomes dominant in the SCS, where the E–W system diminishes or greatly reduces in strength.

The southeastward asthenospheric flow underneath the SCS driven by the ongoing Indian–Eurasian collision represents long-distance kinematics characterized by a spatial scale of several thousand kilometers, which may influence dynamic topography [48] and carry asthenospheric materials southeastward for large distances. Given the coincidence in the timing between the rapid uplift of the Himalayas and the spreading of the SCS [21], certain geological and geodynamic processes in the Tibetan Plateau and the SCS might be connected by the asthenospheric flow since the Oligocene with the former possessing a potential influence on the opening and cessation of spreading of the SCS. In particular, the southeastward extruded flow system could be utilized as an alternative cause for the southward ridge jumps during the seafloor spreading of the SCS occurring at ∼27 Ma and ∼23.6 Ma [18,23].

## CONCLUSIONS

The first systematic investigation of shear wave splitting in the SCS using seismic data from seven broadband OBSs revealed the anisotropic structure and mantle flow fields in the NW and SW sub-basins with an unprecedented resolution and coverage. The dominant mechanism accounting for the observed azimuthal anisotropy is flow in the asthenosphere generated by continental collision between the Indian and Eurasian plates. Frozen rock fabric that was produced during the plate spreading and fossilized in the lithosphere is probably present in the lithosphere which has a fast orientation that is consistent with the direction of the fossil seafloor spreading.

## DATA AND METHODS

The passive source seismic data used for the shear wave splitting analyses were obtained from 16 ocean bottom seismometers (OBSs), among which nine OBSs were operated from September 2019 to July 2020 over a nine-month period, and the other seven OBSs were obtained from a previous array deployed in December 2010 and recovered in early 2011 which have a recording period of approximately three months [40]. After correction for horizontal component orientations, teleseismic core-mantle-boundary refracted shear waves, including PKS, SKKS, and SKS phases, were used to calculate the splitting parameters and constrain the anisotropic signatures, which were obtained based on the transverse energy minimization method [32]. More details are provided in the Supplementary Data.

## Supplementary Material

nwad176_Supplemental_File

## Data Availability

The seismic waveform data used in the study is available upon request.

## References

[1] Le Pichon X . Sea-floor spreading and continental drift. J Geophys Res 1968; 73: 3661–97. 10.1029/JB073i012p03661

[2] Martin-Short R , Allen RM, Bastow ID et al. Mantle flow geometry from ridge to trench beneath the Gorda–Juan de Fuca plate system. Nat Geosci 2015; 8: 965–8. 10.1038/ngeo2569

[3] Long MD , Becker TW. Mantle dynamics and seismic anisotropy. Earth Planet Sci Lett 2010; 297: 341–54. 10.1016/j.epsl.2010.06.036

[4] Conrad CP , Behn MD. Constraints on lithosphere net rotation and asthenospheric viscosity from global mantle flow models and seismic anisotropy. Geochem Geophys Geosyst 2010; 11: Q05W05. 10.1029/2009GC002970

[5] Wolfe CJ , Solomon SC. Shear-wave splitting and implications for mantle flow beneath the MELT region of the East Pacific Rise. Science 1998; 280: 1230–2. 10.1126/science.280.5367.1230 9596569

[6] Faccenda M , Capitanio FA. Seismic anisotropy around subduction zones: insights from three-dimensional modeling of upper mantle deformation and SKS splitting calculations. Geochem Geophys Geosyst 2013; 14: 243–62. 10.1002/ggge.20055

[7] Hall CE , Fischer KM, Parmentier EM et al. The influence of plate motions on three-dimensional back arc mantle flow and shear wave splitting. J Geophys Res 2000; 105: 28009–33. 10.1029/2000JB900297

[8] Fan E , He Y, Ai Y et al. Seismic anisotropy and mantle flow constrained by shear wave splitting in central Myanmar. J Geophys Res 2021; 126: e2021JB022144. 10.1029/2021JB022144

[9] Huang Z , Chevrot S. Mantle dynamics in the SE Tibetan Plateau revealed by teleseismic shear-wave splitting analysis. Phys Earth Planet Inter 2021; 313: 106687. 10.1016/j.pepi.2021.106687

[10] Liu J , Wu J, Wang W et al. Seismic anisotropy beneath the eastern margin of the Tibetan Plateau from SKS splitting observations. Tectonophysics 2020; 785: 228430. 10.1016/j.tecto.2020.228430

[11] Gao Y , Shi YT, Wang Q. Seismic anisotropy in the southeastern margin of the Tibetan Plateau and its deep tectonic significances (in Chinese). Chin J Geophys 2020; 63: 802–16. 10.6038/cjg2020O0003

[12] Zhu T , Guo YX. Lithospheric basal shear traction and strain rate beneath Mainland China constrained by uppermantle shear-wave splitting anisotropy (in Chinese). Chin J Geophys 2021; 64: 2684–700. 10.6038/cjg2021O0328

[13] Kong F , Wu J, Liu L et al. Azimuthal anisotropy and mantle flow underneath the southeastern Tibetan Plateau and northern Indochina Peninsula revealed by shear wave splitting analyses. Tectonophysics 2018; 747–748: 68–78. 10.1016/j.tecto.2018.09.013

[14] Yu Y , Gao SS, Liu KH et al. Characteristics of the mantle flow system beneath the Indochina Peninsula revealed by teleseismic shear wave splitting analysis. Geochem Geophys Geosyst 2018; 19: 1519–32. 10.1029/2018GC007474

[15] Tapponnier P , Peltzer G, Le Dain AY et al. Propagating extrusion tectonics in Asia: new insights from simple experiments with plasticine. Geology 1982; 10: 611–6. 10.1130/0091-7613(1982)10<611:PETIAN>2.0.CO;2

[16] Jia Y , Liu KH, Kong F et al. A systematic investigation of piercing-point-dependent seismic azimuthal anisotropy. Geophys J Int 2021; 227: 1496–511. 10.1093/gji/ggab285

[17] Wang P , Huang C, Lin J et al. The South China Sea is not a mini-Atlantic: plate-edge rifting vs intra-plate rifting. Natl Sci Rev 2019; 6: 902–13. 10.1093/nsr/nwz135 34691951 PMC8291395

[18] Briais A , Patriat P, Tapponnier P. Updated interpretation of magnetic anomalies and seafloor spreading stages in the South China Sea: implications for the Tertiary tectonics of Southeast Asia. J Geophys Res 1993; 98: 6299–328. 10.1029/92JB02280

[19] Li C , Xu X, Lin J et al. Ages and magnetic structures of the South China Sea constrained by deep tow magnetic surveys and IODP Expedition 349. Geochem Geophys Geosyst 2014; 15: 4958–83. 10.1002/2014GC005567

[20] Sun Z , Jian Z, Stock JM et al. South China Sea Rifted Margin. Proceedings of the International Ocean Discovery Program, 367/368. College Station: International Ocean Discovery Program, 2018.

[21] Spicer RA , Su T, Valdes PJ et al. Why ‘the uplift of the Tibetan Plateau’ is a myth. Natl Sci Rev 2021; 8: nwaa091. 10.1093/nsr/nwaa091 34691550 PMC8288424

[22] Sibuet J-C , Yeh Y-C, Lee C-S. Geodynamics of the South China Sea. Tectonophysics 2016; 692: 98–119. 10.1016/j.tecto.2016.02.022

[23] Ding W , Sun Z, Dadd K et al. Structures within the oceanic crust of the central South China Sea basin and their implications for oceanic accretionary processes. Earth Planet Sci Lett 2018; 488: 115–25. 10.1016/j.epsl.2018.02.011

[24] Zhao M , He E, Sibuet J-C et al. Postseafloor spreading volcanism in the central east South China Sea and its formation through an extremely thin oceanic crust. Geochem Geophys Geosyst 2018; 19: 621–41. 10.1002/2017GC007034

[25] Li J , Ding W, Lin J et al. Dynamic processes of the curved subduction system in Southeast Asia: a review and future perspective. Earth-Sci Rev 2021; 217: 103647. 10.1016/j.earscirev.2021.103647

[26] Huang Z , Zhao D, Wang L. P wave tomography and anisotropy beneath Southeast Asia: insight into mantle dynamics. J Geophys Res Solid Earth 2015; 120: 5154–74. 10.1002/2015JB012098

[27] Lin J , Xu Y, Sun Z et al. Mantle upwelling beneath the South China Sea and links to surrounding subduction systems. Natl Sci Rev 2019; 6: 877–81. 10.1093/nsr/nwz123 34691947 PMC8291476

[28] Song W , Yu Y, Gao SS et al. Seismic anisotropy and mantle deformation beneath the central Sunda Plate. J Geophys Res Solid Earth 2021; 126: e2020JB021259. 10.1029/2020JB021259

[29] Argus DF , Gordon RG, DeMets C. Geologically current motion of 56 plates relative to the no-net-rotation reference frame. Geochem Geophys Geosyst 2011; 12: Q11001. 10.1029/2011GC003751

[30] Hua Y , Zhao D, Xu Y-G. Azimuthal anisotropy tomography of the Southeast Asia subduction system. J Geophys Res Solid Earth 2022; 127: e2021JB022854. 10.1029/2021JB022854

[31] Xue M , Le KP, Yang T. Seismic anisotropy surrounding South China Sea and its geodynamic implications. Mar Geophys Res 2013; 34: 407–29. 10.1007/s11001-013-9194-4

[32] Silver PG , Chan WW. Shear wave splitting and subcontinental mantle deformation. J Geophys Res 1991; 96: 16429–54. 10.1029/91JB00899

[33] Zhang S , Karato S. Lattice preferred orientation of olivine aggregates deformed in simple shear. Nature 1995; 375: 774–7. 10.1038/375774a0

[34] Ben Ismail W , Mainprice D. An olivine fabric database: an overview of upper mantle fabrics and seismic anisotropy. Tectonophysics 1998; 296: 145–57. 10.1016/S0040-1951(98)00141-3

[35] Hess HH . Seismic anisotropy of the uppermost mantle under oceans. Nature 1964; 203: 629–31. 10.1038/203629a0

[36] Ando M , Ishikawa Y, Yamazaki F. Shear wave polarization anisotropy in the upper mantle beneath Honshu, Japan. J Geophys Res 1983; 88: 5850–64. 10.1029/JB088iB07p05850

[37] Gao SS , Liu KH, Abdelsalam MG. Seismic anisotropy beneath the Afar Depression and adjacent areas: implications for mantle flow. J Geophys Res 2010; 115: B12330. 10.1029/2009JB007141

[38] Hammond JOS , Kendall JM, Wookey J et al. Differentiating flow, melt, or fossil seismic anisotropy beneath Ethiopia. Geochem Geophys Geosyst 2014; 15: 1878–94. 10.1002/2013GC005185

[39] Silver PG , Savage MK. The interpretation of shear-wave splitting parameters in the presence of two anisotropic layers. Geophys J Int 1994; 119: 949–63. 10.1111/j.1365-246X.1994.tb04027.x

[40] Ruan A , Li J, Lee C et al. Passive seismic experiment and ScS wave splitting in the southwestern subbasin of South China Sea. Chin Sci Bull 2012; 57: 3381–90. 10.1007/s11434-012-5132-0

[41] Liu KH , Gao SS, Gao Y et al. Shear wave splitting and mantle flow associated with the deflected Pacific slab beneath northeast Asia. J Geophy Res 2008; 113: B01305. 10.1029/2007JB005178

[42] Savage M . Seismic anisotropy and mantle deformation: what have we learned from shear wave splitting? Rev Geophys 1999; 37: 65–106. 10.1029/98RG02075

[43] Wang X , Huang H, Xu H et al. The deep thermal structure of the lithosphere in the northwestern South China Sea and its control on the shallow tectonics. Sci China Earth Sci 2021; 64: 962–76. 10.1007/s11430-020-9726-2

[44] Zhang G , Chen L, Jackson MG et al. Evolution of carbonated melt to alkali basalt in the South China Sea. Nat Geosci 2017; 10: 229–35. 10.1038/ngeo2877

[45] Yu Y , Gao SS, Liu KH et al. Mantle transition zone discontinuities beneath the Indochina Peninsula: implications for slab subduction and mantle upwelling. Geophys Res Lett 2017; 44: 7159–67. 10.1002/2017GL073528

[46] Debayle E , Ricard Y. Seismic observations of large-scale deformation at the bottom of fast-moving plates. Earth Planet Sci Lett 2013; 376: 165–77. 10.1016/j.epsl.2013.06.025

[47] Yang BB , Liu Y, Dahm H et al. Seismic azimuthal anisotropy beneath the eastern United States and its geodynamic implications. Geophys Res Lett 2017; 44: 2670–8. 10.1002/2016GL071227

[48] Husson L , Bernet M, Guillot S et al. Dynamic ups and downs of the Himalaya. Geology 2014; 42: 839–42. 10.1130/G36049.1

