# Supplementary material for: Mantle flow underneath the South China Sea revealed by seismic anisotropy
Source: Natl Sci Rev. 2023 Jun 16;10(10):nwad176. doi: 10.1093/nsr/nwad176 (PMC10476890; doi:10.1093/nsr/nwad176)
Supplement: nwad176_Supplemental_File [file nwad176_supplemental_file.docx]

Supplementary Materials for

**Mantle Flow underneath the South China Sea Revealed by Seismic Anisotropy**

Fansheng Kong^1,2,3*^, Rui Gao^2,4^, Stephen S. Gao^3^, Kelly H. Liu^3^, Weiwei Ding^1,2^, Xiongwei Niu^1,2^, Aiguo Ruan^1,2^, Pingchuan Tan^1,2^, Jianke Fan^3^, Shaoping Lu^2,4^, Zhengyi Tong^1^, Liqun Cheng^1^, Wenfei Gong^1^, Yanghui Zhao^1,2^, Jiabiao Li^1,2*^

1 Key Laboratory of submarine Geosciences, Second Institute of Oceanography, Ministry of Natural Resources, Hangzhou, 310012, China

2 Southern Marine Science and Engineering Guangdong Laboratory, Zhuhai, 519082, China

3 Geology and Geophysics Program, Missouri University of Science and Technology, Rolla, MO, 65409, USA

4 School of Earth Sciences and Engineering, Sun Yat-sen University, Zhuhai, 519082, China

5 Institute of Oceanology, Chinese Academy of Sciences, Qingdao, 266071, China

*Corresponding author: Jiabiao Li (jbli@sio.org.cn); Fansheng Kong (kongfs@sio.org.cn)

**Contents of this file**

Supplementary Data and Methods

Figs. S1 to S8

Table S1

Supplementary References

**Data and Methods**

The seismic data utilized for the shear wave splitting analysis in this study were recorded by 16 ocean bottom seismometers (OBSs), of which nine OBSs were operated from September 2019 to July 2020 over a nine-month period, while the other seven OBSs were deployed in December 2010 and recovered in early 2011 with a recording period of about three months (Fig. S1) [1]. Since the OBSs are free-fall, corrections for horizontal component orientations are necessary and conducted based on the elliptical particle motion of Rayleigh waves from large earthquakes with a cut-off magnitude of 5.0 and a focal depth of no deeper than 150 km [2]. The waveforms used to calculate the misorientation of the horizontal components were band-pass filtered using corner frequencies of 1/30 Hz and 1/10 Hz to enhance the signal-to-noise ratio (SNR). The horizontal components of the seven OBSs with a 9-month recording period were reliably corrected (see an example in Fig. S2). By contrast, the three-month deployment has four OBSs with orientations robustly determined.

The teleseismic events used for the shear wave splitting analyses are within the epicentral distance of 120°–180°, 83°–180° and 95°–180° for teleseismic core-mantle-boundary refracted shear waves PKS, SKS, and SKKS (collectively referred to as XKS), respectively. The magnitude is no less than 5.6 for events with a focal depth smaller than 100 km and 5.5 for deeper events [3]. In total, 75 teleseismic events satisfy the above criteria and were used to measure the shear wave splitting parameters (the fast orientation and splitting time), which were obtained by applying the procedure described by Liu and Gao [3] based on the transverse energy minimization method [4]. The time window used for measuring the splitting parameters was initially set as 5 s before and 20 s after the predicted arrival time of the XKS phases computed using the IASP91 Earth Model. The search range for the fast orientation is -90° to 90° with an interval of 1°, while for the splitting time, it is 0 s to 4.0 s with a step of 0.05 s. The error estimation of the resulting splitting measurements was achieved by the 95% confidence region of the *F*-distribution [4]. To enhance the SNR, all the XKS seismograms were initially band-pass filtered with corner frequencies of 0.04 Hz and 1.0 Hz.

Each pair of the splitting measurements was automatically ranked, grouping the measurements into Quality A (Excellent), B (good), C(unusable), and N (null) based on the SNR of the original and corrected radial and transverse components [3,5], the fitness between the corrected fast and slow components and whether the particle motion is elliptical before the correction and becomes linear after the correction. The SNR is defined as $SNR={max\left| A_{(a,f)} \right|}/{max\left| A_{(a-10,a)} \right|}$, where $a$ and $f$ represent the beginning and end of the XKS time window in units of seconds used for measuring the shear wave splitting parameters, $max\left| A_{(a,f)} \right|$ and $max\left| A_{(a-10, a)} \right|$denote the maximum absolute values on the waveform in the time window described as $(a,f)$, and $(a-10,a)$, respectively. The measurements with an automatically determined rank of A, B, or N were then manually checked. During that process, the time window and band-pass filtering frequencies were adjusted to enhance the SNR of the XKS arrivals and exclude non-XKS arrivals.

A total of 26 pairs of well-defined XKS shear wave splitting measurements (Table S1) from 25 teleseismic events (Fig. S3) were obtained at seven OBSs. Most of the events resulted in only one well-defined splitting measurements, which can be attributed to the noisy seismic data and the number of OBSs deployed. Such an observation is also found in the Cascadia Initiative seismometer deployments [6]. An excellent or good measurement (Fig. S4) is characterized by an XKS arrival of good quality on both the original radial and transverse components, and a relatively low SNR on the corrected transverse component (an indicative of the significant energy reduction after the correction), resulting in a linear particle motion. An unusable measurement, by contrast, is typically characterized by either low SNR on the original radial and transverse components in the time window used for the splitting analysis, or the presence of inadequately corrected energy on the corrected transverse component [3,5]. The splitting measurements from the OBSs in the vicinity of the axial fossil ridge of the SW sub-basin are generally characterized by an azimuthally invariant pattern (Fig. S5), while those in the NW sub-basin have a systematic back azimuthal variation with a 90° periodicity (Fig. S6), consistent with the presence of a two-layered anisotropy model (Figs. S7 and S8). As discussed in the body text, the azimuthal variation can be explained by a two-layered anisotropy model with the upper layer related to lithospheric fossilized anisotropy caused by seafloor spreading and the lower layer associated with the present-day NNW-SSE mantle flow.

**Possible Factors** **Accounting for the Low Data Recovery Rate**

The ocean-bottom seismometers (OBSs) are buoyant in the ocean under normal conditions due to the airtight class cabin, which pressure is designed to be lower than the standard atmospheric pressure and thus possesses positive buoyancy. During the deployment, each OBS was attached to an iron heaving-coupling holder with a hook, resulting in negative net buoyancy and positioning the OBS on the seafloor. When recovering an OBS, the anchor is melted after the OBS receives a release command and then the OBS floats to the sea surface. Based on two OBSs that were failed in the initial recovery but recovered later using a manned deep-sea submersible in 2022, a factor accounting for the low recovery rate is the damage of the airtight class cabin, probably due to the large pressure contrast across the class of the cabin when on the seabed of the South China Sea abyssal basin for a long-term deployment. Another possible factor may be associated with the battery which is designed to melt the anchor and probably had run out before the recovery.

**Arguments Against a Mantle Transition Zone and Lower Mantle Origin of the Observed Anisotropy**

The dominant minerals in the lower mantle (bridgmanite and magnesiowüstite) cannot induce the splitting of the sub-vertically traveling XKS phases [7]. Ringwoodite, which constitutes most of the lower MTZ is largely isotropic [8], and in the upper MTZ, the dominant mineral, wadsleyite, can produce azimuthal anisotropy under horizontal shear with a resulting fast orientation normal to the shear direction. According to recent anisotropy tomography investigations [9,10], the anisotropy strengths in the MTZ of the SCS are mostly less than 1%, resulting in a splitting time less than 0.25 s assuming the entire upper MTZ is azimuthally anisotropic. Another possible source of anisotropy is associated with the stagnant slab in the MTZ of the SCS. Metastable olivine that likely exists within subducted plates below the upper mantle can possess azimuthal anisotropy [11]. However, given either the long stagnation time in the MTZ and the thin lithosphere [12], metastable olivine if present in the stagnant slab beneath the SCS, would have a minor contribution to the observed splitting [11]. The arguments suggest that the mantle transition zone and the lower mantle cannot be the main source of the observed NNW-SSE oriented anisotropy.

**Supplementary References**

1. Ruan A, Li J and Lee C *et al*. Passive seismic experiment and ScS wave splitting in the southwestern subbasin of South China Sea. *Chin Sci Bull* 2012; **57**: 3381-90.
2. Stachnik JC, Sheehan AF and Zietlow DW *et al*. Determination of New Zealand ocean bottom seismometer orientation via Rayleigh-wave polarization. *Seismol Res Lett* 2012; **83**: 704-13.
3. Liu KH and Gao SS. Making reliable shear-wave splitting measurements. *Bull. Seismol. Soc. Am.* 2013; **103**: 2680-93.
4. Silver PG and Chan WW. Shear wave splitting and subcontinental mantle deformation. *J Geophy Res* 1991; **96**: 16429-54.
5. Liu KH, Gao SS and Gao Y *et al*. Shear wave splitting and mantle flow associated with the deflected Pacific slab beneath northeast Asia. *J Geophy Res* 2008; **113**: B01305.
6. Martin-Short R, Allen RM and Bastow ID *et al*. Mantle flow geometry from ridge to trench beneath the Gorda–Juan de Fuca plate system. *Nat Geosci* 2015; **8**: 965-8.
7. Tsujino N, Nishihara Y and Yamazaki *et al*. Mantle dynamics inferred from the crystallographic preferred orientation of bridgmanite. *Nature* 2016; **539**:81-4.
8. Mainprice D. Seismic anisotropy of the deep Earth from a mineral and rock physics perspective, in Price, D., and Stixrude, L., eds., Treatise on Geophysics (second edition), Volume 2: Mineral Physics: Amsterdam, Elsevier, 487–538.
9. Huang Z, Zhao D and Wang L. P wave tomography and anisotropy beneath Southeast Asia: Insight into mantle dynamics. *J Geophy Res* 2015; **120**: 5154-74.
10. Hua Y, Zhao D and Xu Y-G. Azimuthal anisotropy tomography of the Southeast Asia subduction system. *J Geophy Res* 2022; **127**: e2021JB022854.
11. Kong F, Gao SS and Liu KH *et al*. Metastable olivine within oceanic lithosphere in the uppermost lower mantle beneath the eastern United States. *Geology* 2022; **50**: 776-80.
12. Li J, Ding W and Lin J *et al*. Dynamic processes of the curved subduction system in Southeast Asia: A review and future perspective. *Earth-Sci Rev* 2021; **217**: 103647.


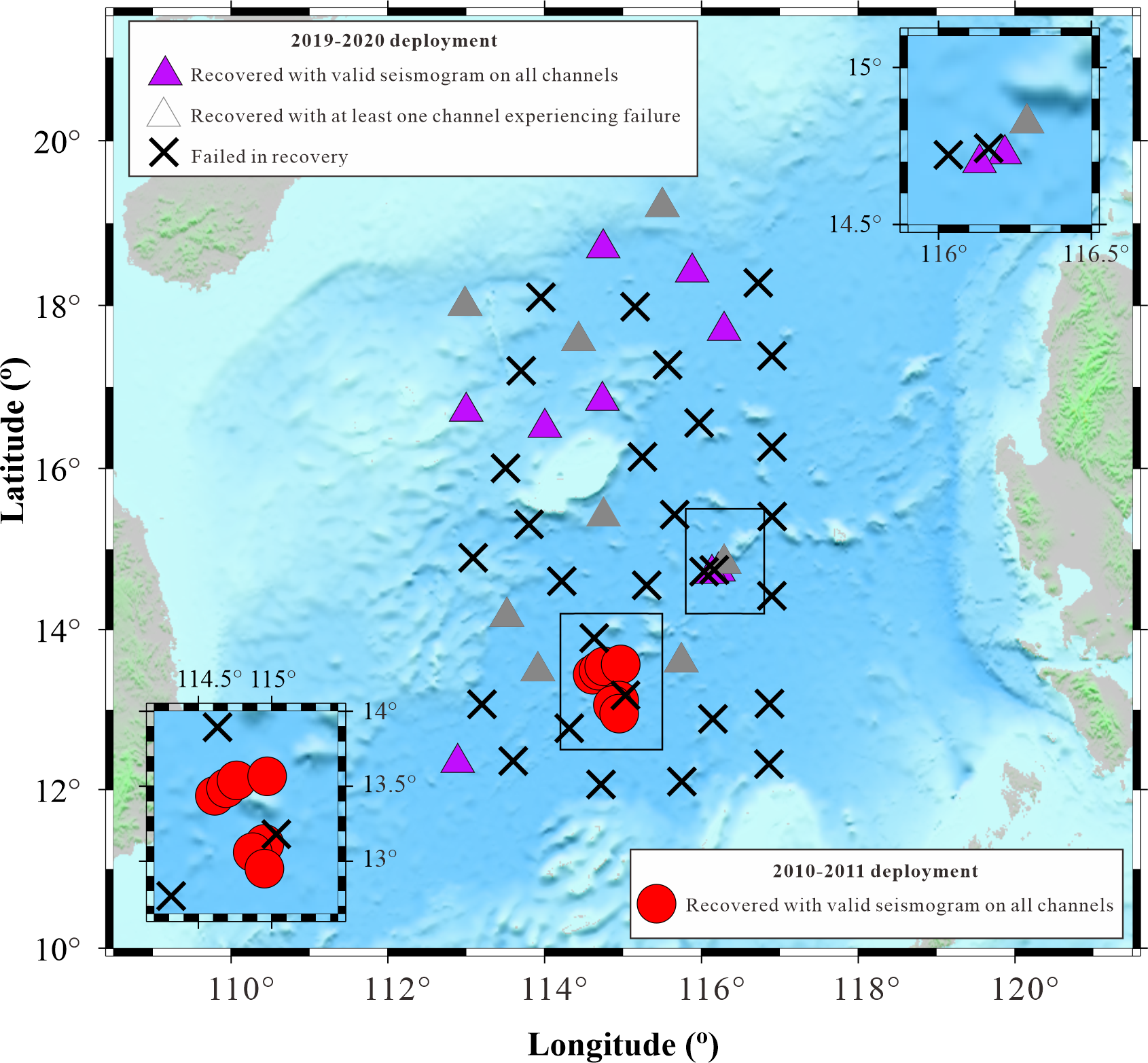


Figure S1. Locations of ocean bottom seismometers (OBSs) used in this study. The triangles represent the OBSs deployed in September 2019 and recovered in July 2020. The red dots denote an OBS array that was deployed in December 2010 and recovered in early 2011.


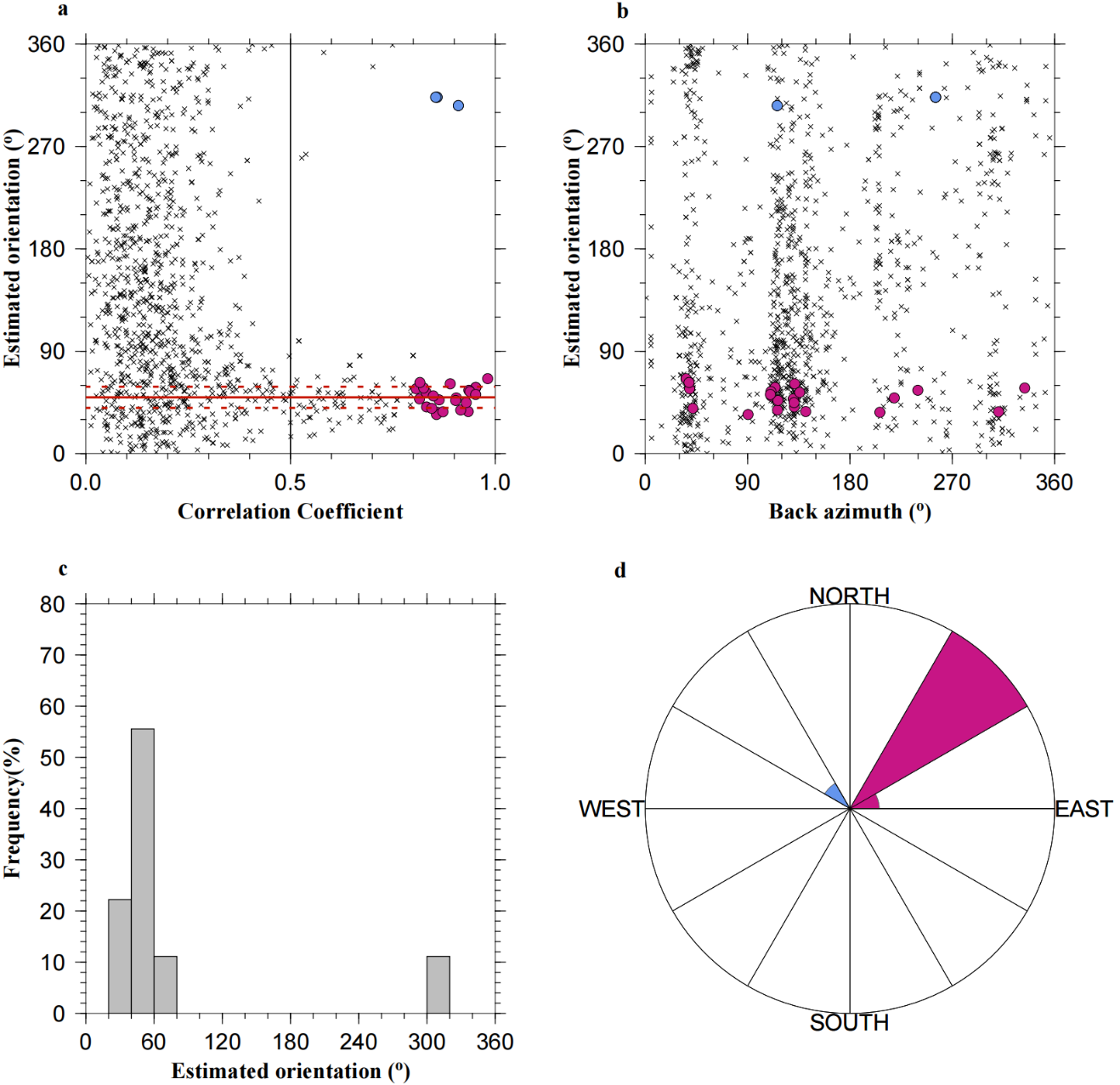


Figure S2. Example of orientation correction for the horizontal components of OBS 28D (14.696°N, 116.134°E). (a) Estimated orientations plotted with respect to the correlation coefficient with values greater than 0.8 shown in dark red or blue. The circular mean (horizontal line) of those measurements shown in the dark red was used for horizontal component corrections, while the outliers (blue dots) were rejected. The dashed lines stand for the standard deviations. (b) Same as (a) but plotted against the back azimuth. (c) Distribution of the estimated orientations in percentage. (d) Rose diagram showing the distribution of the estimated orientations with a correlation coefficient > 0.8.


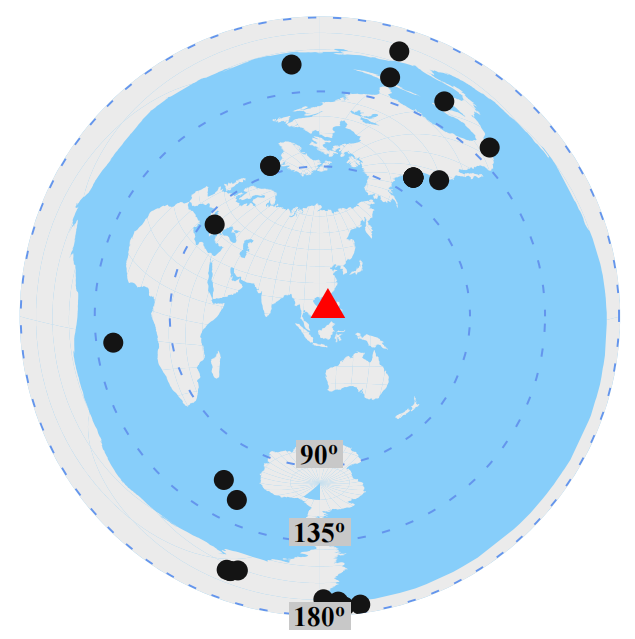


Figure S3. An azimuthal equidistant projection map centered at the study area showing the distribution of earthquakes (circles) that provided one or more good (Quality A or B) shear wave splitting measurements. The numerals represent the distance from the center of the study area (red triangle) in degrees.


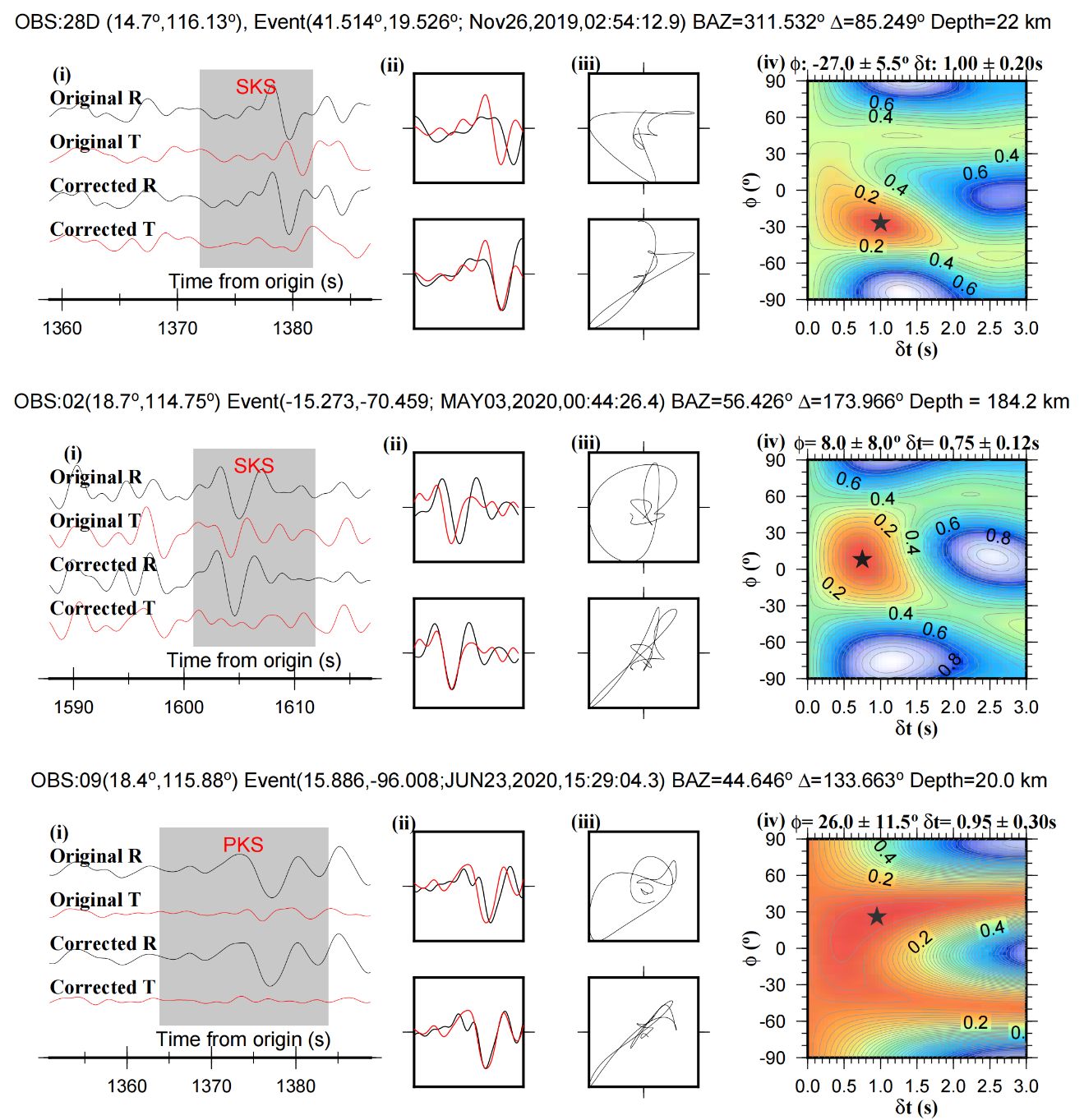


Figure S4. Examples of shear wave splitting measurements using records from OBSs 28D (top) and 02 (bottom). The portion in the shaded area of (i) represents the time window used for the splitting analysis. For each measurement, the plots from left to right show the original and corrected radial (black) and transverse (red) components, original and corrected fast (red) and slow (black) waves, original (upper) and corrected (lower) practical motions, and the normalized misfit function that is computed based on the energy of the corrected transverse component in the time window. The optimal pairs of splitting parameters are marked by stars.


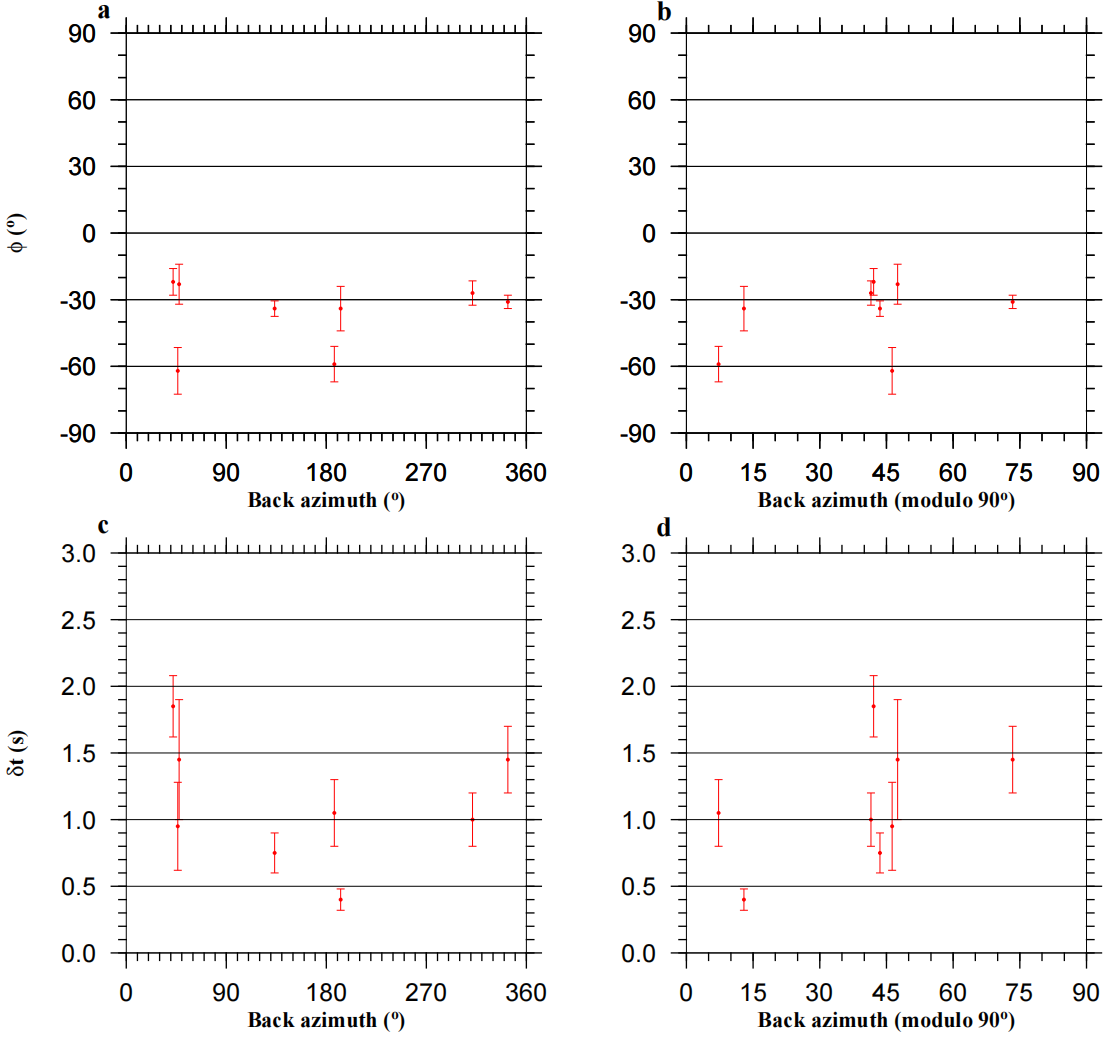


Figure S5. XKS splitting parameters (fast orientation and splitting time) for the OBSs located in the vicinity of the axial fossil ridge of the SW sub-basin plotted against the back azimuth (a and c) and the modulo 90° back azimuth (b and d).


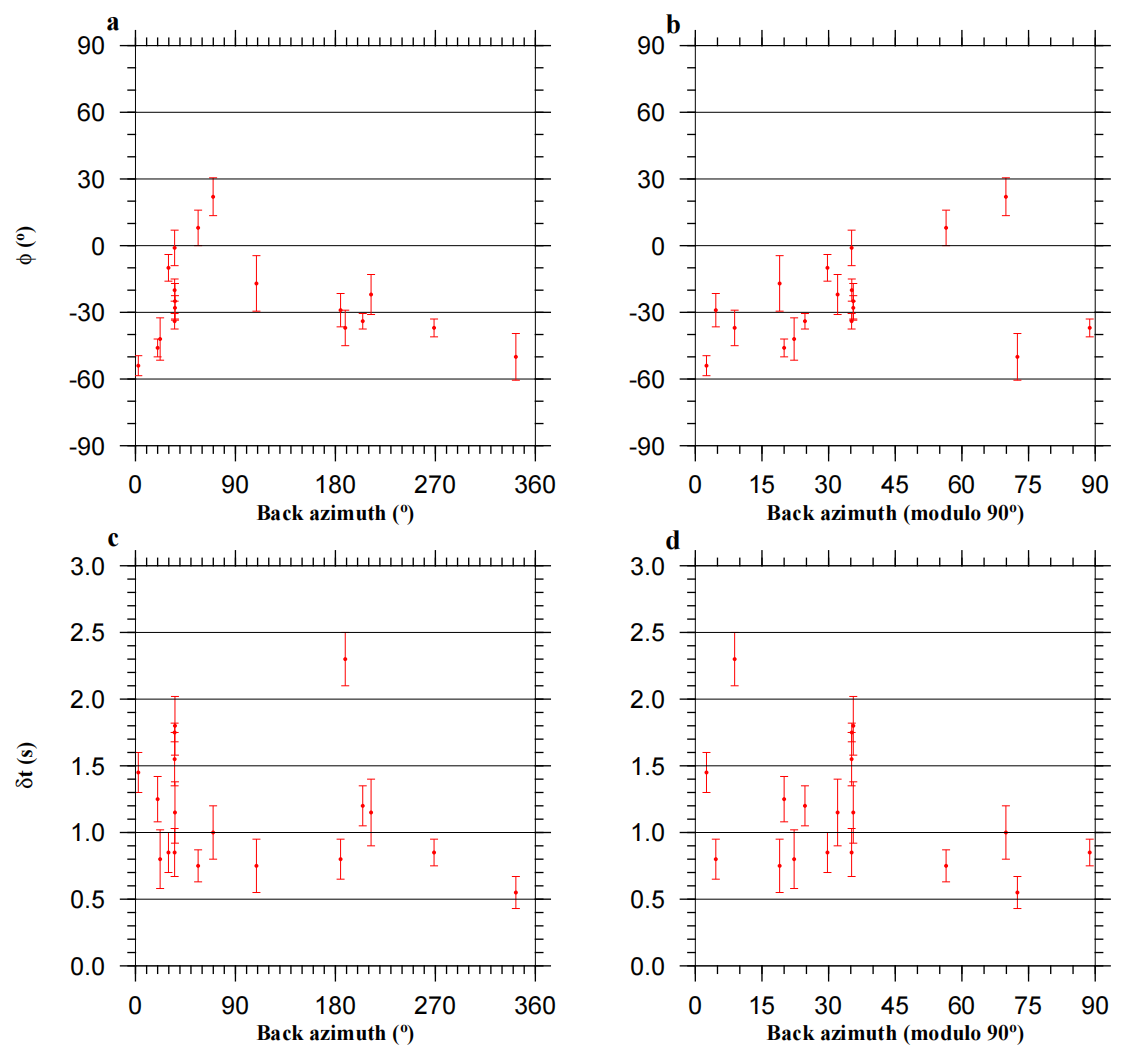


Figure S6. Same as Figure S5 but for the OBSs deployed on the NW sub-basin seafloor and adjacent areas.


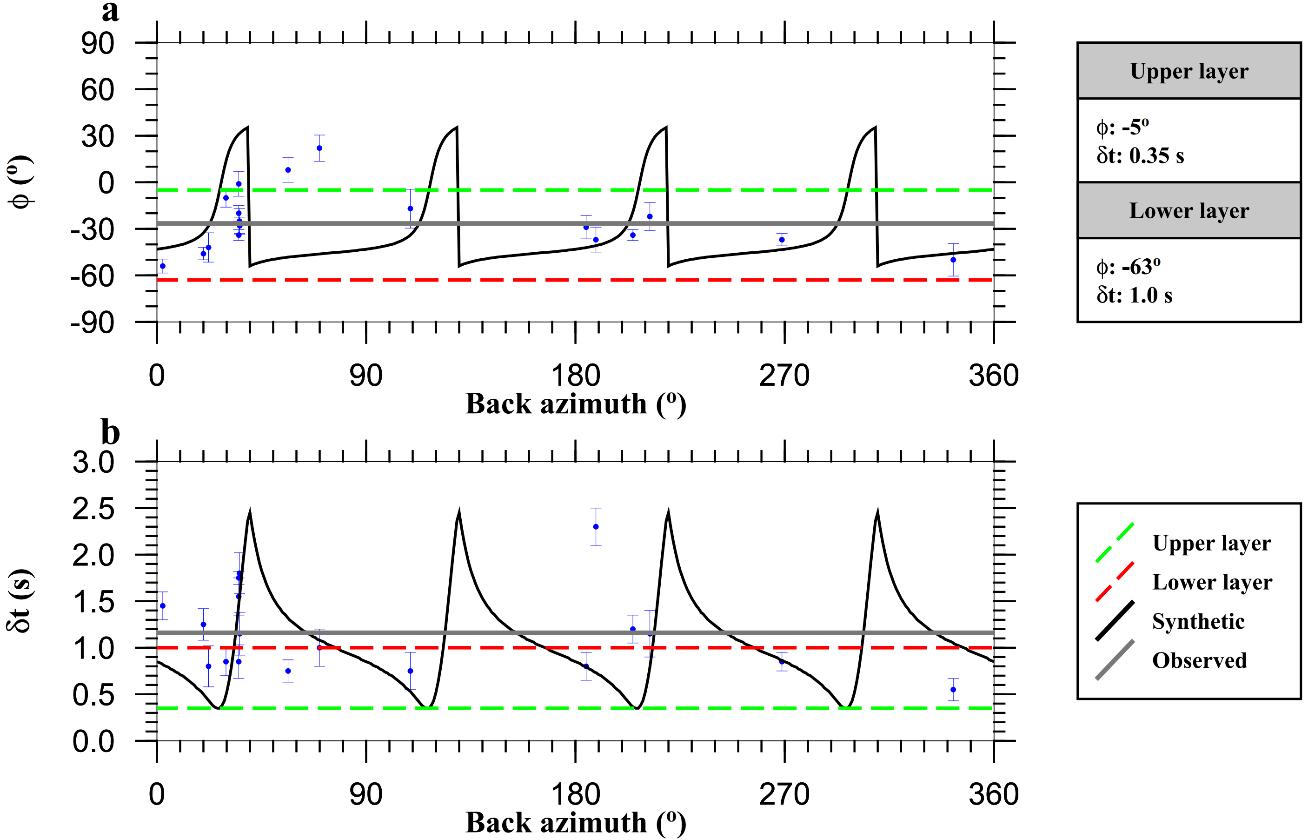


Figure S7. Predicted fast orientations (a) and splitting times (b) plotted against the back azimuth for an anisotropy model composed of two layers with the splitting parameters denoted by the dashed green (upper layer) and red (lower layer) lines. The gray lines represent the circular mean of the fast orientation and the arithmetic mean of the observed individual splitting time measurements obtained in the NW sub-basin, which are shown as blue dots. The anisotropy model has a pair of splitting parameters (-5° or 175°, 0.35 s) for the upper layers and a fast orientation (-63° or 117°) for the lower layer which is consistent with the APM direction. For this model, while the splitting time of the lower layer (dashed red line in b) is close to the station average (solid gray horizontal line in b), the fast orientation of the lower layer (dashed red line in a) differs substantially to the station average (solid gray horizontal line in a).


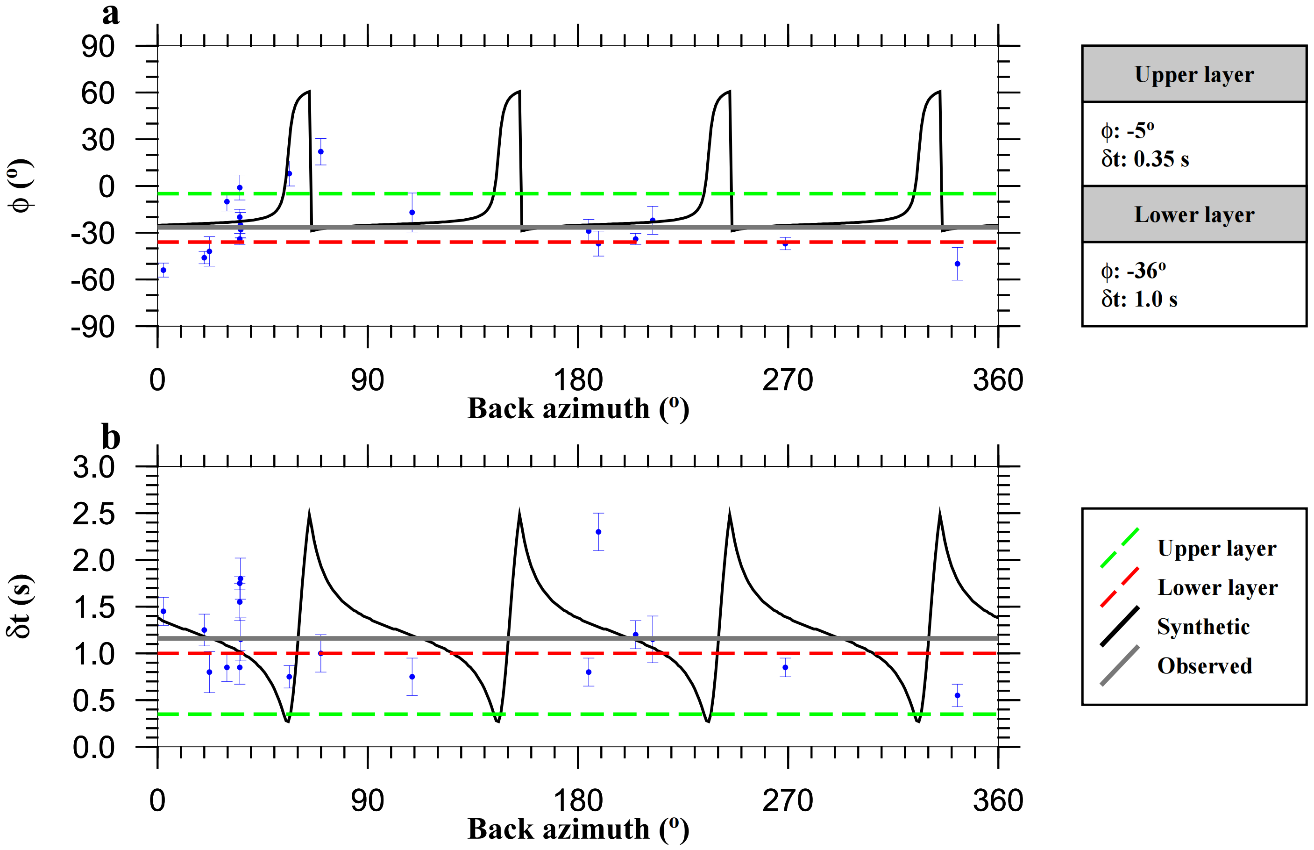


Figure S8. Same as Figure S7 but for an anisotropy model with the lower layer associated with the NNW-SSE extruded flow system, characterized by a fast orientation of -36° (or 144°). Compared to the model shown in Figure S7, the station averaged fast orientation (solid gray horizontal line in a) are much closer to the fast orientation of the lower layer (dashed red line in a). In addition, the theoretical observations (gray curves) fit the observed values better (e.g., the two fast orientation measurements in the azimuthal range of 50−70°).

Table S1. Shear wave splitting measurements obtained in this study. The fields of the table include:

OBS: Ocean-Bottom Seismometer name

Year: Year of deployment

Pha: Phase name

Ev: Event name

Lat: Latitude (°N)

Lon: Longitude (°E)

phi: Fast orientation (°, measured clockwise from north)

phi_SD: Standard deviation of the fast orientation (°)

dt: Splitting time (s)

dt_SD: Standard deviation of the splitting time (s)

R: Rank of the measurements

Origin time: Event origin time

| OBS | Year | Pha | Ev | Lat | Lon | phi | phi_SD | dt | dt_SD | R | Origin time |
| --- | --- | --- | --- | --- | --- | --- | --- | --- | --- | --- | --- |
| 02 | 2019 | SKS | EQ193581903 | 18.70 | 114.75 | 138.00 | 9.50 | 0.80 | 0.22 | B | 2019(358)19:03:52.5 |
| 02 | 2019 | SKS | EQ200070824 | 18.70 | 114.75 | 126.00 | 4.50 | 1.45 | 0.15 | B | 2020(007)08:24:25.3 |
| 02 | 2019 | SKS | EQ200281910 | 18.70 | 114.75 | 134.00 | 4.00 | 1.25 | 0.17 | B | 2020(028)19:10:24.9 |
| 02 | 2019 | SKS | EQ201240044 | 18.70 | 114.75 | 8.00 | 8.00 | 0.75 | 0.12 | B | 2020(124)00:44:26.4 |
| 05 | 2019 | SKS | EQ201731907 | 16.70 | 113.00 | 130.00 | 10.50 | 0.55 | 0.12 | B | 2020(173)19:07:54.8 |
| 09 | 2019 | SKS | EQ193571949 | 18.40 | 115.88 | 155.00 | 8.00 | 1.80 | 0.22 | B | 2019(357)19:49:43.1 |
| 12 | 2019 | SKS | EQ193590336 | 16.83 | 114.74 | 160.00 | 5.00 | 1.75 | 0.07 | B | 2019(359)03:36:01.6 |
| 28D | 2019 | SKS | EQ193300254 | 14.70 | 116.13 | 153.00 | 5.50 | 1.00 | 0.20 | B | 2019(330)02:54:12.9 |
| 34 | 2019 | SKS | EQ201060115 | 12.34 | 112.89 | 146.00 | 10.00 | 0.40 | 0.08 | B | 2020(106)01:15:34.8 |
| 05 | 2019 | PKS | EQ201070804 | 16.70 | 113.00 | 170.00 | 6.00 | 0.85 | 0.15 | B | 2020(107)08:04:37.8 |
| 12 | 2019 | PKS | EQ193370846 | 16.83 | 114.74 | 163.00 | 12.50 | 0.75 | 0.20 | B | 2019(337)08:46:35.8 |
| 12 | 2019 | PKS | EQ200170637 | 16.83 | 114.74 | 143.00 | 4.00 | 0.85 | 0.10 | B | 2020(017)06:37:52.7 |
| 28D | 2019 | PKS | EQ201751529 | 14.70 | 116.13 | 157.00 | 9.00 | 1.45 | 0.45 | B | 2020(175)15:29:04.3 |
| 34 | 2019 | PKS | EQ201751529 | 12.34 | 112.89 | 118.00 | 10.50 | 0.95 | 0.33 | B | 2020(175)15:29:04.3 |
| 02 | 2019 | SKKS | EQ193092052 | 18.70 | 114.75 | 158.00 | 9.00 | 1.15 | 0.25 | B | 2019(309)20:52:01.5 |
| 02 | 2019 | SKKS | EQ193572056 | 18.70 | 114.75 | 179.00 | 8.00 | 0.85 | 0.18 | B | 2019(357)20:56:23.5 |
| 02 | 2019 | SKKS | EQ193590336 | 18.70 | 114.75 | 146.00 | 3.50 | 1.55 | 0.20 | B | 2019(359)03:36:01.6 |
| 05 | 2019 | SKKS | EQ201520509 | 16.70 | 113.00 | 22.00 | 8.50 | 1.00 | 0.20 | B | 2020(152)05:09:38.6 |
| 09 | 2019 | SKKS | EQ193571913 | 18.40 | 115.88 | 152.00 | 5.50 | 1.15 | 0.23 | B | 2019(357)19:13:25.1 |
| 09 | 2019 | SKKS | EQ200200651 | 18.40 | 115.88 | 146.00 | 3.50 | 1.20 | 0.15 | B | 2020(020)06:51:37.8 |
| 09 | 2019 | SKKS | EQ200232111 | 18.40 | 115.88 | 151.00 | 7.50 | 0.80 | 0.15 | B | 2020(023)21:11:28.8 |
| 12 | 2019 | SKKS | EQ200871742 | 16.83 | 114.74 | 143.00 | 8.00 | 2.30 | 0.20 | B | 2020(087)17:42:50.3 |
| 28D | 2019 | SKKS | EQ201990540 | 14.70 | 116.13 | 146.00 | 3.50 | 0.75 | 0.15 | B | 2020(199)05:40:36.3 |
| 28D | 2019 | SKKS | EQ201731907 | 14.70 | 116.13 | 149.00 | 3.00 | 1.45 | 0.25 | B | 2020(173)19:07:54.8 |
| 34 | 2019 | SKKS | EQ200690259 | 12.34 | 112.89 | 158.00 | 6.00 | 1.85 | 0.23 | B | 2020(069)02:59:08.9 |
| 34 | 2010 | SKKS | EQ110010956 | 13.12 | 114.95 | 121.00 | 8.00 | 1.05 | 0.25 | B | 2011(001)09:56:58.1 |
